# Nutritional Influences on Skatole Formation and Skatole Metabolism in the Pig

**DOI:** 10.3390/ani2020221

**Published:** 2012-05-02

**Authors:** Raffael Wesoly, Ulrike Weiler

**Affiliations:** Institute of Animal Husbandry and Animal Breeding, Behavioral Physiology of Farm Animals (470a), University of Hohenheim, Garbenstr. 17, 70593 Stuttgart, Germany; E-Mail: wesoly@uni-hohenheim.de

**Keywords:** skatole, pig, feeding, skatole physiology, inulin, raw potato starch, boar taint

## Abstract

Skatole is a tryptophan metabolite with fecal odor. Skatole and the testicular steroid androstenone are regarded as the main compounds leading to ‘boar taint,’ a sex-specific odor from pork taken from entire males, as elevated concentrations of both substances may be found in adipose boars tissue. High skatole concentrations in adipose tissue are the result of a complex process, which includes microbial formation in the colon, absorption, metabolism and accretion in fat. Several of these steps leading to high skatole concentrations are influenced by feed components and additives. The present paper discusses the mechanisms by which effective feeding strategies and feed additives exert their influence in the prevention of high skatole concentrations in adipose pig tissue.

## Abstract (executive summary)

Skatole is a tryptophan (TRP) metabolite with fecal odor. Together with the testicular steroid androstenone it is regarded as a main determinant of boar taint, even if elevated concentrations of skatole occur occasionally in gilts and barrows. Skatole concentrations in adipose tissue result from a complex process, which includes the availability of TRP and the presence of specialized bacteria in the gut in need of TRP for energy production, as well as absorption, transport and accumulation of skatole in adipose tissue. Several steps of this process are influenced by diet and specific feed compounds. In the present paper the current knowledge about physiological mechanisms of skatole dynamics is summarized. Additionally mechanisms are discussed, by which effective feeding strategies and feed additives exert their influence in the prevention of high skatole concentrations in adipose pig tissue. It was concluded that the most effective measures are those which influence several steps of skatole formation. Despite the numerous studies carried out in the field of skatole physiology, interesting aspects still need clarification, such as the effect of adipose tissue turnover. Reliable control of skatole accretion in fat of boars is one of the main prerequisites for pork production with entire males.

## 1. Introduction

Boar taint is one of the major problems occurring in meat production of entire male pigs [1]. The two main compounds contributing to boar taint are androstenone (5α-androst-16-ene-3one, [2,3]), a steroidal pheromone produced in the Leydig cells of the testis, and skatole (3-methyl-indole, [4]), a product of microbial degradation of tryptophan (TRP) with fecal odor [5]. Skatole can be found in several species including ruminants, where it is mainly formed in the rumen, and monogastrics, such as the pig, where it is mainly formed in the colon [6]. In the pig, skatole has gained considerable scientific interest and is discussed as one of the principal compounds contributing to the off-odor of meat from entire males, but elevated concentrations occur occasionally in females and barrows [7]. Whereas the physiology of androstenone formation has been studied extensively and environmental and genetic influences are known [8,9], the physiological mechanisms of skatole formation, absorption and its metabolism within the pig are less clear. While androstenone concentration is only moderately influenced by nutrition [9,10,11,12,13], various feeding strategies are known to influence the concentrations of skatole in adipose tissue. This topic has been the subject of several scientific reviews during the last decade [9,14,15]. As a lot of new studies are available, it is the aim of the present paper to describe current knowledge about the mechanisms leading to skatole formation and accretion in adipose tissue of pigs. Based on these mechanisms, an overview on possible feeding strategies or nutritional manipulations of skatole formation is summarized and their efficiency discussed.

## 2. Physiology of Skatole Formation and Deposition in Adipose Tissue

### 2.1. Biochemical Pathway and Microbial Activity

Skatole results from a multistep degradation of TRP by microbial activity, mainly in the hind gut of the pigs [16]. Analogously, many other metabolites such as indole are formed which may also contribute to off odor in pork [17]. 

**Figure 1:**
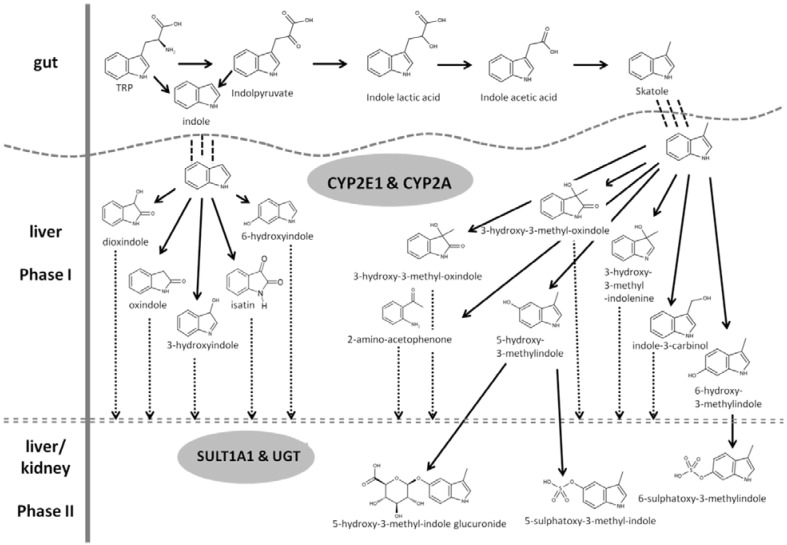
Formation of skatole (3-methyl-indole) and indole from TRP in the gut and further metabolism via phase 1 and phase 2 enzymes, (black arrows: known pathway; broken arrows: assumed pathway) [46,54,55,57,62].

The reaction mechanism of TRP degradation is limited under the anaerobic conditions of the intestinal tract to reductive processes at the 3-position of the TRP indole ring structure, yielding the terminal products skatole and indole [16]. Whereas many bacteria are able to metabolize tryptophan to indole and indole acetic acid (IAA), the key precursor of skatole, only a few specialized gut bacteria, mainly from the Clostridium and Bacteroides genera, can catalyze the steps from IAA to skatole [18,19,20]. It was estimated that these bacteria represent less than 0.01% of the total intestinal flora [21]. In case of skatole, intermediate products are indole-3-lactic acid and indole-3-pyruvic acid, resulting from the deamination of TRP, which is decarboxylated to IAA, and then further decarboxylated to skatole (see Figure 1, upper part) [16,20]. This metabolic pathway has been identified in *Lactobacilllus* sp. strain 11201 [22] but also in *C. drakeii* and *C. skatologenesis* using deuterium labeled TRP or IAA through *in vitro* studies with bacteria from swine manure [20]. In this study, adding TRP together with glucose reduced the amount of skatole and increased IAA with both types of Clostridia. Similarly, the addition of TRP to cell cultures rich in *C. scatologenes* did not necessarily increase skatole formation [23]. Such data suggest that the microbial skatole forming activity is reduced, if alternative energy substrates are available, as further discussed below (see Section 3.3).

In two more recent studies which compared the microbial community in the colon of Jinhua and Landrace pigs, *C. aminophilum* was also suggested to affect skatole production in the Jinhua breed [24]. The same authors published an *in vitro* study with porcine gut microbes, which points to a contribution of *C. disporicum* to skatole formation [25]. A summary of formation and metabolism of skatole and indole in the pig is given in Figure 1. 

### 2.2. Anatomical Sites of Skatole Formation

TRP degradation to indole and skatole starts in the proximal part of the colon. In contrast to earlier studies [26,27], which already revealed elevated concentrations of indole in the distal part of the small intestine and the cecum, the majority of more recent studies point to a later onset of TRP degradation to indole and skatole [24,28,29]. In one of the feeding studies [28], only traces of free tryptophan were detectable (<100 µmol/kg digesta) in the stomach but increased in the small intestine. Dividing the small intestine into three parts, elevated concentrations above 200 µmol TRP/kg digesta were already found in the proximal part, increasing to maximum concentrations (about two-fold) in the middle part. In the distal part, TRP concentrations decreased to values comparable to those of the proximal part. No conversion of TRP to indole or skatole was measurable from the stomach to the distal part of the small intestine. Low concentrations of skatole and indole were found in the cecum, but maximum concentrations of IAA and IPA (indolic pyruvic acid) were observed, which decrease continuously along the colon [28]. Correspondingly, almost linearly increasing concentrations of skatole and indole are observed. Maximum concentrations of both compounds are found either in the distal colon or the rectum [14,24,28,29]. 

Absorption of skatole and indole occurs along the colon and both substances are transferred to the liver via the portal vein. Total daily absorption rates of skatole were estimated between 820 and 365 µmol skatole, depending on the diet [28]. The daily absorption of indole was reported to be about threefold higher and ranged between 2,999 and 929 µmol [28]. The concentrations of skatole in portal blood, peripheral blood and feces is highly correlated within individuals, but that of indole is not, suggesting that the amount of skatole absorbed is proportional to the amount produced [27,28]. Such correlations were less obvious in one study if several individuals were included or between animal correlations were calculated [27,28], as individual differences in metabolism of skatole seem to exist [30]. 

The direct infusion of skatole into the cecum of pigs via a cannula in the ileo-cecal junction in 4 h intervals for three days increased skatole concentration in feces only slightly and excretion rates via feces were below 5 %. An absorption rate above 90% of the amount of skatole, which is already available in the proximal part of the colon, was discussed [31]. Under physiological conditions, however, only a small amount of the total skatole production is formed in the proximal part of the colon, as shown above [28]. 

Thus, in a similar experimental approach using TRP infusion into the cecum, an appearance rate in the portal vein of 70% of the skatole formed along the colon was calculated. In contrast to a former study [32], this percentage seemed to be independent on the total amount of skatole synthesized [14].

### 2.3. Origin of Tryptophan as a Precursor for Skatole Synthesis

The origin of TRP needed for the microbial synthesis of skatole is controversial [24,27]. Whereas in ruminants, the addition of TRP to the diet regularly leads to increased skatole formation [33,34], the effect of TRP in the diet of the pig is less clear [35,36,37]. Diets with low prececal protein digestibility were reported to increase skatole production [5,14,38], whereas feed supplements with synthetic L-TRP below or above the requirements (1.0 g/kg DM to 1.91 g/kg DM) were not effective in systematically changing skatole concentrations in the feces of growing pigs [37]. It was postulated that free TRP is mainly absorbed in the small intestine and is therefore not available for microbial metabolism in the colon. Studies aiming to influence the rate of mitosis and apoptosis in the small intestine experimentally showed that gut cell debris is a major source of tryptophan for the microbial skatole formation [39,40]. 

Changes in diet may lead to a reorganization of intestinal mucosa and thereby increase the amount of cell debris, as most obvious after weaning in piglets [41]. A clear increase in skatole formation after weaning was clearly shown for piglets of different sex and weaning ages, which, however, was accompanied by an age dependent decrease in SULT1A1 (see Section 2.4; [30,42]). Feed additives (antibiotics and Chinese herbs), which reduced the number of pathogenic bacteria in the intestine were effective in reducing atrophy of villi in piglets after weaning [41] 

The direct infusion of 4.9 mmol TRP into the cecum of pigs was effective in increasing skatole concentrations in portal blood. The increase started after 2 h, reached maximum values up to five-fold concentrations after 6 h to 10 h and decreased thereafter [14]. The total conversion of infused TRP to skatole was estimated, based on portal blood concentrations. It ranged between 26% for animals given a low fiber diet and 6% for animals given a high fiber diet. Most of the infused TRP was converted to indole (69% and 35% respectively). In animals fed with high fiber diet a higher amount of indole-propionic acid (IPA) was measured, compared with animals on a low fiber diet (16 *vs.* 7%) [14].

Thus a generally lower proportion of degradation products of TRP was observed if more fiber was fed simultaneously. Under such conditions, only 57% of the cecally-infused TRP was metabolized to one of the three compounds (skatole, indole, IPA), whereas in animals on a low fiber diet the total amount of infused TRP was degraded to one of the three substances. It was concluded that in the case of a high fiber diet, more TRP is used for the microbial synthesis of protein instead of microbial energy production via TRP degradation [14]. Other factors such as colon motility, fecal transit time, secretory rates, osmolality in the colon which are influenced by the physiological and psychological status of the host may also have a significant role [16]. 

The transfer of skatole and indole from gut to blood is probably due to passive diffusion, as there is no skatole carrier protein known [43].

### 2.4. Metabolism of Skatole in Liver and Kidney

Skatole and indole are transported by the portal vein (V. porta) to the liver, where most of the indole derivatives are metabolized by specific enzymes. Small amounts of indoles, which are absorbed in the distal colon or rectum, can bypass the liver and are transferred via the vena cava caudalis directly into the peripheral bloodstream [10,27]. The liver metabolism is highly effective and skatole concentrations of the portal vein may be reduced severely in the liver (up to 90%), as was obvious from parallel measurements in the hepatic vein [32,44]. Based on these data, a half-life for skatole in blood of one hour was calculated [44]. 

Even if skatole and indole are mainly degraded in the liver [45,46,47], a contribution of other organs, such as the kidneys, as similarly shown for androstenone degradation, may be assumed [48]. The knowledge on hepatic metabolism of skatole and indole has been reviewed recently [8,9]. In brief, hepatic degradation of indoles can be divided in two distinct steps: an oxidative step, phase 1 metabolism, and a conjugative step, phase 2 metabolism. Responsible enzymes are various cytochrome P450 isozymes, which are known to play a predominant role in drug and xenobiotic metabolism. Two specific enzymes, CYP2E1 and CYP2A were identified as major enzymes of the phase 1 metabolism of skatole [47,49]. A minor contribution of other P450 isozymes to the skatole phase 1 metabolism was discussed, but is less clear [50,51,52,53,54]. During phase 1 metabolism skatole is degraded to seven intermediate products (see Figure 1, lower part). The main enzymes of phase 2 metabolism, SULT1A1 (sulfotransferase) and UGT (uridine-di-phosphate-glucuronosyltransferase) further modify these seven compounds mainly by increasing their hydrophilic properties, adding either a sulfate or glucuronyl group to the molecules [44]. Phase 2 metabolism thus results in a variety of terminal products, where 6-sulfatoxy-skatole, sulfated or glucuronic conjugates of 5-hydroxy-3-methylindole and 3-hydroxy-3-methyloxindole are predominant [55,56,57]. During phase 2 metabolism, the water solubility of the skatole metabolites is increased, facilitating excretion via urine [11,58]. Enzymes of phase 2 metabolism are mainly located in the liver, but can also be found other tissues, such as kidneys and lungs [48]. The main products of phase 1 and phase 2 metabolism are shown in Figure 1 (lower part).

The activity of the phase 1 enzymes is modulated by several gonadal steroids. It was shown that the addition of physiological concentrations of androstenone led to a significant reduction in the activity of CYP2E1 and CYP2A in hepatic microsomes from pigs [47,53], while the addition of 17ß- and 17a-estradiol only revealed such an inhibiting effect in supraphysiological concentrations [59]. Similarly, a higher enzyme activity of these two enzymes in the hepatic tissue of barrows was reported [60], compared to activity in tissue of boars. It was concluded that the lower skatole concentrations in adipose tissue of barrows were due to these differences. Higher levels of enzyme expression were reported for barrows and immunocastrates when compared to boars, but this high expression level did not always result in high enzyme activity, suggesting the influence of additional factors [50]. This hypothesis was further supported by an *in vitro* study [53], where gender specific effects of a pre-incubation with androstenone on the activity of CYP2E1 and CYP2A in liver microsomes were reported. In this study, enzymes derived from liver microsomes of female pigs were not inhibited by the pre-incubation with androstenone, up to 15 ng/mL, whereas the same enzymes from male liver samples exhibited a decreasing activity after the addition of 15 ng androstenone/mL or 0.5 ng estradiol/mL to the *in vitro* system. The addition of testosterone at a dose of 5 ng/mL was without effect in both genders. A slight increase (10%) in CYP2A activity was induced by adding 0.5 ng 17ß-estradiol/mL to liver microsomes from female pigs [53]. 

This effect of sex hormones on hepatic skatole degrading enzyme activity offers an explanation for the regularly reported higher concentrations of skatole in blood and tissue of boars compared to sows, gilts and barrows [7,45,61].

### 2.5. Accretion of Skatole in Adipose Tissue

As a lipophilic substance, skatole is accumulated in adipose tissue if blood levels of skatole are elevated for a prolonged period. Similarly, skatole concentrations in adipose tissue drop within days, if skatole formation in the colon is reduced due to feeding measures, such as inulin supplementation as described below [10]. These changes occur more rapidly than in the case of the even more lipophilic steroid androstenone [10]. 

Adipose tissue differs within and between animals in its fatty acid turnover and in its stability according to its fatty acid composition. Main factors influencing fatty acid composition are the anatomical site (e.g., organ fat *vs.* subcutaneous or intramuscular fat), the overall degree of fatness of an individual or breed and the diet in monogastrics [63,64]. It is well established that leaner pigs have a lower ability to synthesize fatty acids combined with a greater mobilization, which results in adipose depots with more unsaturated lipids [65,66]. 

A relationship between skatole deposition and the turnover rate of adipose tissue has not yet been proven, but breed comparisons in skatole concentrations suggest such an influence. Lean breeds, such as the Pietrain, revealed generally lower skatole and androstenone concentrations than the fatter Large White breed [67]. Similarly, the distribution of skatole within the carcass is in accordance with the amount of saturated fatty acids of the specific adipose tissue. Both are higher in flare than in belly fat, and higher in belly fat than in adipose tissue from the neck [68,69,70], suggesting again that adipose tissue with a higher amount of SFA and lower turnover accumulates more skatole. Specific studies on fat turnover and skatole dynamics however need to be conducted.

### 2.6. Summary of the Physiological Mechanisms Leading to Elevated Skatole in Adipose Tissue

**Figure 2:**
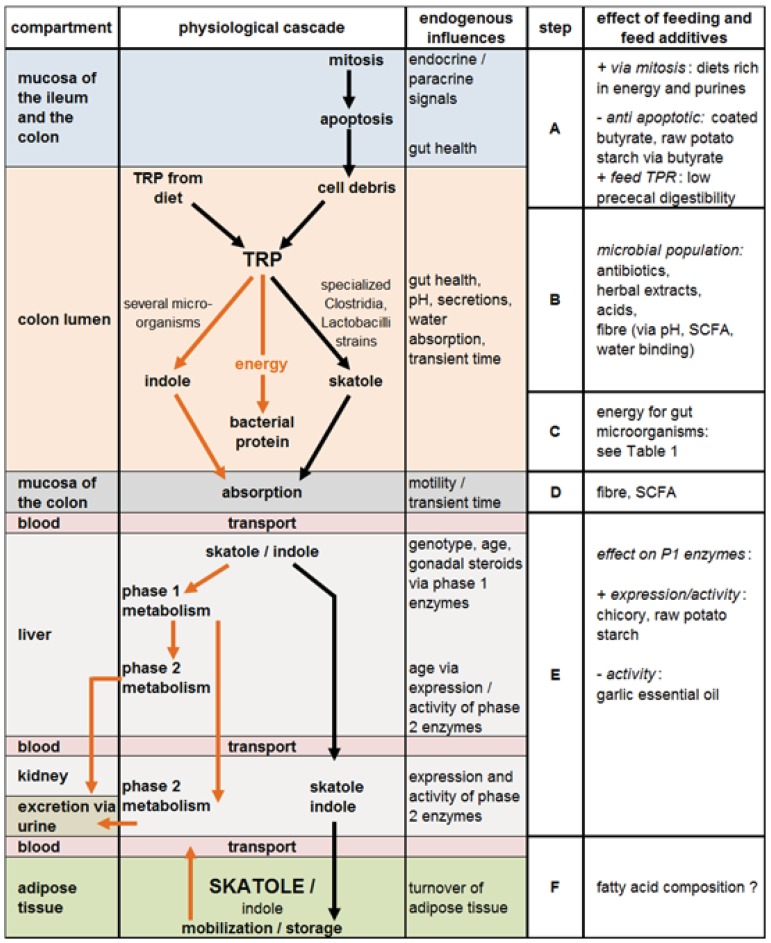
Cascade of physiological events leading to skatole formation, further metabolism and accumulation of skatole in adipose tissue. Steps correspond to Sections 3.1 (A) to 3.6 (F). The right column shows where distinct feeding influences exert their effects. Black arrows: steps leading to high skatole concentrations; brown arrows: skatole reducing or neutral conditions.

The mechanisms leading to elevated concentrations of skatole in adipose tissue of pigs are summarized in the Figure 2. High concentrations of skatole require (A) a high amount of TRP with low prececal digestibility or cell debris for microbial degradation in the colon, (B) specialized microbes for skatole synthesis, (C) insufficient alternative energy sources for microbial activity, so that the metabolism of TRP to skatole occurs instead of the synthesis of bacterial protein, (D) a high absorption rate, such as in the case of a long transient time of digesta, (E) a reduced degradation of skatole in phase 1 metabolism of liver and phase 2 metabolism in liver and kidney and (F) the deposition in adipose tissue, which requires continuously high concentrations of skatole in peripheral blood and a low turnover of adipose tissue.

This model allows a discussion of the mode of action of feed components on the complex mechanisms leading to a change of skatole concentrations in adipose tissue. 

## 3. Effects of Diet and Feed Additives on Skatole Formation, Absorption, Metabolism and Accretion in Adipose Tissue of Pigs

As shown in Figure 2, high or low concentrations of skatole in adipose tissue are the result of several independent steps, such as the formation, absorption, metabolism and accretion in adipose tissue. In the following section, feed components and feed additives which influence skatole dynamics are summarized and related to the physiological steps (Figure 2), contributing to skatole accumulation in adipose tissue. Feeding techniques, such as liquid feeding *vs.* dry feeding, exerted only minor effects on skatole formation [71,72]. Additionally, effects of feed intake behavior (feed intake rate, duration of meals) may explain some variability in skatole formation [73] but these aspects are not further discussed in this review.

### 3.1. Effects of Diet on TRP Availability in the Colon and Consequences for Skatole Formation (Step A, Figure 2)

The effect of TRP availability was investigated by feeding diets with high and low prececal digestibility. It was obvious, as stated earlier, that diets with free TRP supplementation had only a low effect on skatole formation [37], whereas feeding animals using products with a 20% lower prececal digestibility of TRP (blood, meat and bone meal) than the control diet led to increasing skatole concentrations in adipose tissue [38]. 

In a series of studies, Claus and co-workers changed the TRP availability in the colon by influencing the mitosis and apoptosis rate in the small and large intestine [29,39,40,70,74,75]. These studies utilized an increased supply of energy and purines to increase mitosis and consequently apoptosis [39,40], and also the application of either coated butyrate [74] or butyrate-producing feed components, such as raw potato starch [29,70,75]. The increase of mitosis—as confirmed by histological evaluations—resulted in the postulated increase in skatole in feces, blood and adipose tissue, whereas the inhibition of apoptotic processes in the colon, as in a part of the studies confirmed by histochemical evaluation of the mucosa, led to low skatole concentrations in all compartments analyzed [29,70,74,75]. In contrast, the addition of coated butyrate in low amounts from 1.2 to 1.5% was without effect on the skatole formation in the colon and its accretion in adipose tissue as well [76,77].

### 3.2. Effects of Diet or Feed Supplements on Microbial Population in the Gut and Consequences for Skatole Production (Step B, Figure 2)

As skatole is a product of microbial degradation, several strategies are documented to influence the microbial ecosystem in the gut of pigs to reduce skatole forming microbes. Such strategies include antimicrobial compounds or feed additives leading to changes in intestinal pH. It has been shown earlier that skatole-producing bacteria are favored under acidic conditions (pH 5.0), whereas indole-forming bacteria predominate if the pH was increased to 8.0 [5,18]. 

The most radical approaches to influence the microbial population within the gut are the applications of antibiotics such as Tylosin, Virginiamycin and Bacitracin and are mainly limited to studies during the late 90s [18,71,78,79]. Such rude treatments of the intestinal microbial ecosystem resulted in decreasing skatole concentrations in adipose tissue and/or feces if increasing concentrations of these substances were applied [18,71,78], whereas low doses were without effect on skatole in adipose tissue [79]. More recently, studies were published in which gut microbes were influenced via feeding of organic acids with weakly antibacterial properties (e.g., 1.0% formic acid, 0.85 % benzoic acid, 0.85% sorbic acid, corresponding to 0.85% of pure acid) or components, which are metabolized by gut microbes to terminal SCFA [76,77]. Digesta samples for microbiological examinations were taken from proximal jejunum, colon descendens, and rectum. Pigs fed organic acids had significantly lower levels of coliforms, enterococci, and lactic acid-producing bacteria in all sampling sites of the gastro-intestinal tract. Formic acid had a stronger antibacterial effect on coliforms than benzoic or sorbic acids in the small intestine. It was concluded that supplementing diets with different organic acids reduces the number of coliforms, enterococci, and lactic acid-producing bacteria in the gastrointestinal tract. Even if there were no differences in levels of skatole or indole in colon descendens among pigs fed organic acid-supplemented diets, compared to a control diet, plasma skatole levels were significantly reduced in pigs fed diets containing formic acid or benzoic acid [76]. The levels of skatole in entire male pigs however were not reduced in a similar study [77].

The antimicrobial activity of selected plant extracts, such as Chinese medicinal herbs or feed additive derived from Sanguinaria canadensis, essential oil components from herbs and spices or tannin-rich preparations, may offer a strategy to reduce skatole formation [41,80,81,82]. The addition of tannin-rich extracts of several plants inhibits either microbial activity directly or by reducing the availability of proteins for bacterial metabolism. Such extracts were effective to reduce skatole *in vitro* [82], and *in vivo* in ewes [83]. A study with essential oil components from herbs and spices revealed that carvacrol, thymol, eugenol and trans-cinnamaldehyde influence the microbial ecosystem and fermentation pattern in the gastrointestinal tract of pigs [81]. Under conditions of the jejunum, carvacrol and thymol showed very similar and non-selective antimicrobial properties. In cecal simulations, carvacrol, thymol and trans-cinnamaldehyde were equally effective while eugenol had an effect only on coliforms, but the effects of skatole, however, were not investigated. The addition of Chinese medicinal herbs to the diets of piglets after weaning had a clear effect on the bacterial population in the gut, reduced the occurrence of diarrhea and led to an increased villus height in the small intestine, suggesting reduced availability of cell debris. The effect on skatole formation was not measured [41].

A feed additive derived from Sanguinaria canadensis contains large amounts of sanguinarine, an alkaloid with known *in vitro* antimicrobial properties [84]. It was postulated that this alkaloid inhibits the activity of the amino acid, decarboxylase, which is involved in the synthesis of indole and skatole [85] and its application to prevent skatole formation has been claimed by a patent (EP 0581926, US 20030190344). Convincing studies with growing pigs, however, were not published. Approaches to control skatole formation by the addition of plant extracts may be attractive, as they reveal antimicrobial properties at very low concentrations [41,81] but need more systematic investigations.

In contrast, the addition of different sources of fermentable carbohydrates to the diets of growing pigs led to reduced skatole formation or accretion in adipose tissue in several studies. These studies are summarized in Table 1. Easily fermentable carbohydrates are thought to exert two main effects: One is the change of intraluminal pH within the colon, which is assumed to influence the microbial ecosystem of the colon. The other is related to their function as a microbial energy source and further discussed in Section 3.3.

Thus, *in vitro* results after the addition of 0.5–1.5% fructooligosaccharides (FOS), which revealed an increase in indole formation but not in skatole, were discussed as a result of a changed microbial ecosystem via feed-induced pH changes [86]. Similarly, differences in the intraluminal pH were reported after feeding oat-based diets *versus* barley-based diets [87]. Oat-based diets led to a higher pH and concomitantly higher indole concentrations when compared to the barley-based diets. As skatole concentrations were not changed by the treatments, it was postulated that indole-producing bacteria favored the higher pH, as similarly shown in *in-vitro* studies [5,18].

Even if the mechanisms were not clarified in detail, differences in the microbial populations were reported after the supplementation of diets of growing entire male pigs during the last weeks before slaughter with either chicory inulin (see Section 3.3) or potato starch in increasing amounts (0 to 15% in pellets). A reduction of enterobacteria counts in colon descendens and rectum was shown only for the inulin-containing diets with a tendency towards reduced levels of Enterococcus spp. in colon descendens and rectum. The concentrations of skatole in the digesta were also reduced in the case of ≥6% of inulin supplementation, but not in the potato starch groups as further discussed below. The addition of raw potato starch in feed pellets was without effect on the microbial population [88].

The effect of a high protein diet may be associated with higher prevalence of the Clostridiaceae family and the Clostridium genus, which are predominantly proteolytic microbes. Furthermore, a reduced prevalence of the order Clostridiales, particularly the predominately saccharolytic microbes, especially butyrate producers, have to be expected (for review see [89]). Consequences for skatole formation, however, were not studied in detail but may favor skatole production.

### 3.3. Dietary Manipulations Resulting in Higher Energy Availability for Microbial Activity and Their Consequences for Skatole Formation (Step C, Figure 2)

Most of the studies which were effective in reducing skatole formation and deposition in adipose tissue increased energy availability and shifted microbial metabolism from proteolytic to saccharolytic. This feeding strategy was suggested in an early study in which inulin supplementation and bicarbonate were effective in significantly reducing skatole in pig feces. The decrease in adipose tissue was confirmed by two consecutive biopsies one week apart [10]. The importance of fiber as such an energy source was finally proven by the elegant experiment described in part already in Section 2.3 of this review, reporting differences in the conversion rate of TRP infused into the cecum to skatole, if animals were either on a diet with a low or high amount of fiber [14]. Such a diet influenced both the production of skatole in the hindgut and the absorption of skatole to the portal blood. With the low fiber diet, the hindgut bacteria transformed 26% of the infused tryptophan into skatole, resulting in a significant increase of skatole concentration in the portal blood, compared to 6% in the case of a high fiber diet. In the case of the high fiber diet, only 57% of the infused TRP was converted into indoles, whereas in animals on low fiber diets, the total amount was degraded to indoles [14]. It might be assumed that in the case of the high fiber diet, the remaining TRP was used for the synthesis of bacterial protein as similarly discussed after the application of fructooligosaccharides (FOS) to reduce skatole concentrations [86]. 

Feed supplementation for two weeks with 10% of either raw potato starch, FOS, such as inulin, lupines and barley hull meal was significantly effective in reducing skatole concentrations in daily blood samples of pigs, when compared to a control diet (barley, soya). The addition of either palm cake, coconut cake or sugar beet pulp at the same concentration revealed no significant effect [14]. Since then, numerous studies have been carried out to evaluate the minimum dosage of such fiber additive to reduce skatole formation and thus the risk of skatole tainted pork. The most prominent fiber sources are either chicory inulin or raw potato starch (RPS) added to the diet during the last weeks before slaughter. Such studies are summarized in Table 1.

Diets supplemented with RPS proved to be effective in reducing skatole concentrations in hind-gut and adipose tissue, if at least 20% was added to the diet [29,75,90]. Reducing this amount of RPS to 10% of the diet, in contrast to blood, resulted in no significant reduction of skatole in adipose tissue being observed [18,91]. In contrast to the effect of raw potato starch powder, the addition of the same substance before pelleting did not influence skatole concentrations [88], since the process of gelatinisation at high temperatures increased prececal digestibility [92]. Therefore this application type was chosen as a control, when specific effects of raw potato starch on skatole formation were investigated [29,75].

Similar effects to those of RPS on skatole concentrations in fat or feces could be achieved by adding at least 6% inulin via the addition of chicory root extract to the diet [88,93]. However, adding 5% of a chicory extract, containing 66% inulin, had no effect [91]. It has to be kept in mind that these additives may exert their effect also via other mechanisms of skatole formation, e.g., via butyrate formation, as described previously for TRP availability (Section 3.1), or microbial population (Section 3.2). Additional effects on hepatic skatole metabolism are discussed later (Section 3.5). 

**Table 1:** Feed components used to influence skatole formation and accretion via fiber supplementation in recent studies and their effectiveness as influenced by the amount in the diet and duration of application. Amounts of feed components and inulin are given as percentage of the fresh matter (DM: dry matter; n.d.: not determined; +: significant decrease in skatole concentration (*p* < 0.05); (+): tendency for decrease in skatole concentration (*p* < 0.10); - no significant change in skatole concentration).

feed component	% in diet	% effective ingredient in diet	feeding duration (d)	decrease of skatole (p < 0,05) in			[ref.]
				adipose tissue	blood	digesta/feces	
**feed components with main effective ingredient inulin**							
**dried chicory ** **roots**	**10**	n.d.	**16**	-	-	n.d	[11]
	**10-13.3**	**3.6–4.7**	**7–14**	-	-	n.d	[94]
	**25**	**13.95**	**7**	+	+	n.d	[12]
			**14**	+	+	n.d	
			**42**	+	+	n.d	
**crude chicory roots ** **(25% DM)**	**56.3**	**6.9**	**28**	+	+	n.d	
			**42**	+	+	n.d	
			**63**	+	+	n.d	
**dried chicory ** **root extract**	**3**	**1.8**	**14**	-	n.d	n.d	[13]
		**2.1**	**28**	-	n.d	-	[93]
		**2.1**	**30**	-	n.d	-	[88]
	**6**	**3.6**	**14**	+	n.d	n.d	[13]
		**4.2**	**28**	+	n.d	+	[93]
		**4.2**	**30**	+	n.d	+	[88]
	**9**	**5.4**	**14**	+	n.d	n.d	[13]
		**6.3**	**28**	+	n.d	+	[93]
		**6.3**	**30**	+	n.d	+	[88]
**Inulin**	**16.3**	**15.5**	**42**	+	+	n.d	[12]
**Jerusalem artichoke**	**8.1**	**4.2**	**7**	-	n.d	+	[95]
	**12.2**	**6.3**	**7**	(+)	n.d	+	
**other feed components**							
**raw potato starch**	**10**	n.d.	**28–42**	-	n.d	n.d	[91]
	**10**	n.d.	**14**	n.d.	+	n.d.	[18]
	**20**	n.d.	**14**	+	n.d	n.d	[90]
		n.d.	**14–21**	+	n.d	+	[75]
	**30**	n.d.	**14–21**	+	n.d	+	
**lupin seeds**	**10**	n.d.	**28–42**	-	n.d	n.d	[91]
	**25**	n.d.	**30**	+	n.d	+	[88]
**sugar beet pulp**	**10**	n.d	**28**	n.d	+	-	[28]
	**15**	n.d	**fattening period**	-	n.d	n.d	[96]
	**20**	n.d.	**30**	+	n.d	n.d	[97]

Four types of carbohydrate sources, sugar beet pulp (SBP), rye grass hay (RYE), alfalfa hay (ALF) and FOS were compared in their effects on the *in vitro* metabolism of L-tryptophan to skatole and indole by a mixed bacterial population from the large intestines of pigs [25]. Addition of SBP or FOS showed a significant inhibitory effect on skatole formation and relative production rate. 

In contrast, the addition of both types of hay significantly increased skatole concentrations and the relative rate of skatole production after 24 h. The authors postulated that in the presence of fermentable carbohydrates these are used preferentially as an energy source by the intestinal bacteria, leading to a reduced protein catabolism [25,98]. In the case of a relatively higher fermentable RYE and ALF, the carbohydrate source is rapidly depleted (within 15 h), which in turn leads to a switch in the metabolism to proteolysis thereafter [25]. The availability of the slow fermentable SBP may provide a carbohydrate source for the bacteria over the whole investigation period of 24 h, preventing a metabolic switch to proteolysis of the skatole producing microbes. These results suggested that reduced concentration of skatole observed in the presence of SBP and FOS may be caused by an overall decreased rate of tryptophan degradation [25].

### 3.4. Effect of Feeding on the Absorption Rate of Skatole (Step D, Figure 2)

The effect of transient time on skatole absorption was more discussed than investigated. Positive correlations between dry matter content of feces and skatole concentrations in feces (µg/g DM) were reported for growing pigs [37]. It was discussed for other species that the formation of short-chain fatty acids, methane, CO_2_ and other products from the bacterial metabolism of inulin may also contribute to increased peristalsis in the whole gastro intestinal tract, leading to changed conditions for bacterial fermentation and intestinal absorption in general [99]. 

Studies with the addition of complex clay minerals to the diet of pigs to bind skatole to prevent the absorption were published, but were not successful in all studies [91,100]. Similar approaches with more advanced additives, which should bind skatole and thus reduce the absorption rate, were carried out as *in vitro* studies, were not proven to be effective *in vivo*, but may be promising [101]. 

### 3.5. Effects of Feeding on Degradation of Skatole in the Liver and Kidney (Step E, Figure 2)

Various components of feed may either increase or inhibit the activity of skatole degrading enzymes of the phase 1 metabolism. The most impressive effect was obtained by adding garlic essential oil at a dose from zero to 2.15 g/kg to the feed. Concentrations of skatole and indole in adipose tissue increased after 57 days of feeding 2.15 g/kg garlic essential oil from 39.6 ng/g skatole and 35.8 ng/g indole in the control group to 1,001.5 ng/g skatole and 972.5 ng/g indole in the garlic supplemented group. It was suggested that the sulfur containing compounds (allyl di- and tri-sulfides) had an inhibitory effect on the CYP-dependent skatole metabolism, thereby decreasing clearance of skatole and indole from the blood, since similar effects have been observed in other species [38,83,102].

Stimulatory effects of feed components on enzyme expression, as characterized by measuring either transcription through mRNAs or the enzyme activity, as characterized via enzymatic conversion rates, were reported for enzymes of the phase 1 metabolism in the liver [52,97]. For example, activity or expression of phase 1 enzymes were increased after the addition of dried chicory [52], sugar beet pulp [97] and potentially raw potato starch [90] to the diets. Similar effects were not described for any of the phase 2 enzymes [11]. 

Interesting differences between expression levels, protein concentrations and final enzyme activities were reported [52], since a 10% increase in mRNA for CYP1a2, CYP2a and CYP2E1 was measurable after addition of 10% dried chicory to diets. However, protein concentrations and enzyme activity were only higher for CYP1a2, and CYP2a in that experiment. A similar effect on the metabolism of androstenone after feeding chicory roots via an influence on hepatic 3ß-HSD (3ß-hydroxy steroid dehydrogenase) has been recently reported [11]. However, this feedstuff seems to exhibit a highly variable effect due to varying concentrations of inulin in the dried roots [94].

Linseed within a diet was also shown to reduce skatole concentrations in adipose tissue of pigs [103]. It was suggested by the authors that myristicin (a benzodioxole compound found in linseed) induces hepatic P450 enzymes such as CYP2E1, which in turn leads to an increased degradation of skatole [104]. 

### 3.6. Effects of Feeding on Accumulation of Skatole in Adipose Tissue (Step F, Figure 2)

Only a few studies with parallel monitoring of skatole concentrations in blood and adipose tissue biopsies were carried out [10]. Only very little data are available, which allow conclusions to be drawn about the effect of fatty acid composition of feed and adipose tissue on skatole in relation to tissue turnover rate. For example, feeding a linseed diet which provides an elevated amount of PUFA led to a lower skatole accretion. However, this result was not discussed with turnover phenomena [103]. A contribution of dietary manipulations on skatole concentrations via the influence of an increased fat turnover may be discussed and needs further investigation. 

## 4. Conclusions

The reliable control of skatole accretion in the fat of boars is one of the main prerequisites for pork production with entire males. Skatole formation in the gut is the result of several independent conditions, leading to elevated TRP availability for specialized bacteria in need of this substrate for energy production. Even high skatole formation in the colon does not necessarily elevate skatole in adipose tissue, as absorption varies and the metabolism mainly in the liver may degrade sufficient amounts of skatole to ensure low adipose tissue concentrations. Only if the production and absorption exceed the ability of the liver to degrade skatole via mainly phase 1 enzymes, then skatole may accumulate in adipose tissue. This accumulation may be further modified by adipose tissue turnover; however, this aspect needs further clarification.

Nutritional effects on almost all steps of the process leading to skatole accumulation in adipose tissue have been described. The effectiveness of a feeding intervention to reduce skatole concentrations in adipose tissue is high, if the feed additive affects several steps of skatole synthesis and accumulation. Thus the most efficient feeding interventions are actually the addition of inulin or raw potato starch, which influence the microbial ecosystem, probably via the intestinal pH, the energy availability for bacteria and the TRP availability via possible anti-apoptotic effects. Inulin seems to be effective at lower doses than raw potato starch. The antimicrobial activity of selected plant extracts or essential oil components from herbs and spices may offer an additional strategy to reduce skatole formation. 
